# On the Appearance of the Yellow-Fever in the Quarantine Harbour of Marseilles

**Published:** 1821-11

**Authors:** 


### *On the Appearance of the Yellow-Fever in the Quarantine Harbour of Marseilles.*

WE have been favoured with a copy of the official Report, made by the Intendants of Health at Marseilles, to the foreign consuls resident in that city, respecting the appearance of yellow-fever in the lazaretto. It is dated the 20th of September last; and we submit a literal translation of it to our readers, as a document by no means devoid of interest.—A. B. G.

---

SIR,—The yellow-fever having made its appearance in various ports of Spain, and it being deemed unsafe, under the circumstances of the case, to admit any ship or vessel coming from those ports, except in the quarantine harbours of France, it was almost natural to expect that the disease would, sooner or later, make its appearance in some of those establishments.

On the 13th instant, Captain Moldtz, a Dane, commanding the brig *Niseline*, coming from Malaga in ballast, sent to the lazaretto of the island of Pomergue,* a cabin-boy ill of the fever, who died on the succeeding day, the 14th instant.

On the 15th, two seamen, belonging to Captain Demoro, a Sardinian, commanding the brig *St. George*, coming from the Aigles (in the province of Murcia), died; the one at Pomergue, and the other on his way from Pomergue to the lazaretto. These two individuals had scarcely complained of being indisposed on the preceding day, and the rest of the crew were in perfect health.

On the same day (the 15th), I. S. Chiozzoti, an Austrian, commanding the brig *Comte de Goes*, last from Cyprus, and whose ship was anchored in the harbour of Pomergue, at a short distance from that of Captain Moldtz, was sent to the lazaretto, with two of his crew and a *guardian of health*,†—all of them ill. The captain died on the evening of the same day.

Four sailors from the same brig were likewise sent to the lazaretto on the 16th; and the corpse of the second *guardian of health*, who had died on-board the brig the same day, was brought on-shore at the same time. One of the four seamen died on the 18th instant.

On the same day (the 16th), the mate of the ship *Catherine* (French), commanded by Captain Simon, coming from Havre, but having touched at Malaga, fell ill, and was sent to the lazaretto.

It results from the above facts, that four vessels at anchor in the harbour of the island of Pomergue are infected with a con-

---

* Off Marseilles.

† An officer sent on-board every vessel performing quarantine, who remains till the expiration of the time, to ensure a strict compliance with the regulations of the magistrates of health.

tagious disease, which has been unanimously declared, by all the physicians and surgeons, to be the yellow-fever.

This disease may have been imported from Spain by the three vessels coming from that country; but it is evident that it must have been contracted at Pomergue, by the brig commanded by Captain Chiozzoti, which had left Cyprus since the 13th of June, and had continued in the most perfect health up to the 12th of September; when it must have become affected by the malignant influence of the miasmata from the neighbouring vessel of Captain Maldtz.

The state of the patients, however, on-board the vessel belonging to Captain Chiozzoti exhibited different symptoms, on which the physicians and surgeons have deemed it necessary to fix their attention, and which would seem to indicate a complication of typhus with the yellow-fever.

The four infected vessels have been completely segregated from the moment that the nature of the disease was clearly ascertained. The rest of the ships performing quarantine in the harbour of Pomergue, although distant enough from the former to escape all chance of contagion, and enjoying up to the present moment the best state of health, will not be admitted to perform the termination of their quarantine at the chain of the harbour, until after they shall have been kept and watched a certain length of time at the anchorage of Frioul. Vessels arriving from any other port than those of Spain, before the four infected vessels shall have been duly and completely purified, shall begin their quarantine at the anchorage of Endome.

These measures of insulation and segregation, intended to check the effects of contagion and prevent its further progress, are enforced with the concurrent assistance of all the civil and military authorities of the town.

Already they have been followed by those satisfactory results which the experience of other times led us to expect. The contagion seems arrested, and its victims, up to this moment, are limited to the number of six; while, of the seven which are yet ill, five are in a fair way of recovery. No occurrence worthy of notice has taken place on-board the infected vessels since the 16th; and the means employed for their purification seem to have had a beneficial effect on the health of the remaining part of the crew.

The situation of our quarantine establishments is such, that all communication between them and the town, by which the health of the inhabitants might be endangered, is rendered impossible.

Every circumstance leads us to believe that we shall have the satisfaction of informing you shortly of the entire restoration of these establishments to health.

In the mean while, we think it necessary to give you a candid account of the real state of things, with a view to check the false or exaggerated reports which might reach you; hoping that the rigid precautions which we shall never fail to adopt for the preservation of the public health, will, on the present occasion, be crowned with the same success with which Providence has deigned to reward our humble efforts at other times.

We have the honour to be, with the highest consideration,

(Signed) P. B. ROUX,
BONNIN,
CROZET D'ALAYE.

*20th September*, 1821.

To the Consul of His Britannic Majesty resident at Marseilles.

To the above official Report we beg leave to subjoin extracts from the consul's letters of the 25th of September.

"A Report has been received of a case, suspected to be one of yellow-fever, having occurred in the city. A man, employed with several others in clearing the harbour of quarantine, was, on the yellow-fever appearing among the shipping, sent, with his companions, to the lazaretto, to perform a quarantine of eight or ten days; and, on its expiration, he returned to his house in town. This man was attacked, on the 23d of September, with a fever which bore the suspicious symptoms of being the yellow-fever. He was sent back immediately to the lazaret; and his companions, and all the inmates of his house, have since been sent there. He continues ill; but the precise nature of his disease is not yet officially announced.

"The epidemy, in other respects, wears a favourable appearance. No new case has occurred since the 16th instant; and, of the seven men last reported ill, six are getting well, and one died.

(Signed) "J. TURNBULL, *Consul.*"

"*1st October.*

"The Health-office have issued a decree, prohibiting the admission into this port of all foreign vessels coming from the coasts of Spain, and of French vessels under similar conditions.

"The individual who was taken ill of fever under suspicious symptoms in this city, as mentioned in my former dispatch, [see the above communication,] is expected to recover. None of his family, or of the attendants upon him, have been indisposed.

(Signed) "J. TURNBULL, *Consul.*"

